# Accounts of a New and Fatal Epidemic That Prevailed Recently in Mississippi and Tennessee

**Published:** 1847-08

**Authors:** B. J. Hicks, B. F. Taylor

**Affiliations:** Vicksburg, Miss.; Whiteville, Hardeman County, Tennessee


***Accounts of a New and Fatal Epidemic that Prevailed Recently in Mississippi and Tennessee.*** By B. J. HICKS, M. D., of Vicksburg, Miss., and B. F. TAYLOR, M. D., of Whiteville, Hardeman County, Tennessee.—(It is a remarkable coincidence that we should have received about the same time the following communications, evidently describing the same curious disease, as it prevailed at localities nearly 300 miles apart. It will be seen that Dr. White gives no name to the strange affection; whilst Dr. Hicks calls it *myelitis petechialis*. We are satisfied it is the *cerebro-spinal meningitis*, which prevailed during the last year in Ireland and some parts of Europe—an interesting account of which may be found among our Foreign extracts in the present number, taken from Ranking's Half-Yearly Abstract, vol. 2, No. 2, p. 192. Upon enquiry, we learn that several cases of a similar nature were seen in this city in the early part of the spring; and on reflection, we are inclined to think it was the same disease that killed so many of the 2d regiment of Mississippi Volunteers as they passed through this place in January last. We think it evident that neuralgic and spasmodic affections have increased greatly in the south within a few years past.—Eds.)

Editors of the New Orleans Medical and Surgical Journal.

Gentlemen:—In the neighborhood of this village we have had an epidemic that has proven to be one of the most formidable, probably, in the records of medicine. It appears to have been more prevalent near Hatchie river, than in any other section of the country. The disease has been confined principally to children between the age of six and fifteen years. The attack is ushered in with cold chilly sensations, after which moderate heat of surface, pain commencing between the shoulders, extending to the occipital region, rigidity of the posterior cervical muscles, retracting the head considerably backward, as in *tetanus*. Delirium supervenes in an hour or two, contraction of the pupils of the eye, dilatation of the one eye sometimes, with contraction of the other, ptosis of the eyelids, ecchymoses under the eye and on the body, rigidity of the abdominal muscles, spasmodic twitchings of the flexors of the extremities, and a disposition to keep the legs in motion from side to side alternately. A difficulty in expanding the lungs, breathing through the nostrils principally, constipation of the bowels, and sometimes retention of urine. Stertorous breathing comes on, and death soon closes the scene—such are the general symptoms of the disease. It terminates its course in from fifteen to seventy-two hours. I have known one case to terminate as late as the twelfth day.

Almost every method of treatment has been devised and carried into effect; bleeding, emetics, cathartics, cold douche, cupping, mercurials, blisters; after which opium, quinine, and stimulants. The system appears to be so excessively shocked that the recuperative powers are not sufficient to sustain the tottering fabric. The necessary chemical change is not carried on in the lungs; the blood is not decarbonized; consequently, from the phenomena manifested, we are led to the conclusion that death is produced principally by *asphyxia*.

I will simply detail one case, the particulars of which will be found highly interesting, illustrating clearly and conclusively the pathology of the disease.

*Case.*—I was called in consultation with my friend Dr. Durham, an old practitioner, in the night of the 30th March, to see a servant girl, aged 11 years, the property of John H., Esq. She was taken on Sunday evening, the 28th, and was seen the following morning by Dr. Durham. She was then delirious, conjunctivæ injected, pupils contracted, retraction of the posterior cervical muscles, twitchings of the flexors, rigidity of the abdominal muscles, hurried respiration, pulse full, but compressible, constipation of the bowels. A mercurial cathartic was ordered, and a blister to the nape of the neck, extending over the occipital region.

29th. Delirium ceased; there is still retraction of the muscles; medicine produced two evacuations; has passed a small quantity of urine; says she feels better. Ordered calomel and pulv. doveri.

30th. This evening I saw her the first time. Intellect clear, complains of pain in the hypochondriac region, retraction of posterior muscles, pupils contracted, the eye looks rather dull, tenderness on percussing the 2d and 3d dorsal vertebræ, restlessness, pulse quick and compressible, no fever, tongue of a dull red or purple colour around the borders, coated with a thin yellow fur and fissured, respiration quicker than natural, performed principally by the abdominal muscles, dulness on percussion over the right hypochondriac region, auscultation detected slight engorgement of the lungs, no purgative since yesterday. I advised that she should be cupped over the spine pretty extensively, and to have an active mercurial cathartic.

31st. The cupping relieved the muscular retraction; skin feels pliant, though there is increased heat of the scalp. In the course of the night it was thought proper by Dr. Durham to administer ʒi. tinct. opii camph. to relieve excessive abdominal pain. Bowels not moved; the anodyne had the desired effect. She says that she is better. Ordered ol. ricini and ol. terebinth., to be repeated in four hours, should it not operate.

When we were in the act of leaving, the mother came out from the cabin and desired us to come back and see her, as she was much worse. She stated that the patient had asked for a drink of water a few minutes previously, complaining on lying down of pain in the abdomen, and evacuated her bowels in bed. The pupils were contracted, pulse depressed, breathing stertorous. Death closed the scene in a few minutes.

I examined the body in presence of Dr. Durham, and the following were the appearances.

*Sectio. Cadaveris*, 18 *hours after death.*—The body was not thin, and there was a quarter to half an inch of fat covering the abdominal muscles.

*Head.*—The posterior integuments were swollen, both pupils were dilated. On removing the calvarium, a considerable amount of blood flowed from the sinuses of the dura mater. The arachnoid membrane adhered with moderate firmness to the surface of the convolutions.

While the brain was being removed, some two or three ounces of serum escaped from the ventricles, being a clear and transparent colour. The weight of the brain was not ascertained, but it appeared heavier than usual. The surface of the convolutions was much flattened. The base of the brain bore evident marks of inflammation. The membranes covering the medulla oblongata and the cerebellum, the right lobe more especially, were thickened and opaque, adhering likewise pretty firmly to the fissures of Sylvius. The membranes at the base were unusually vascular, but the substance of the brain itself was not altered very much in colour or consistence. The membranes, more particularly, around the third nerve of the right side, were thickened and more vascular than natural. On examining the superior surface of the brain and separating the two hemispheres slightly, they gave way inferiorly. This was ascertained to arise from softening of the lower part of the middle lobe of both hemispheres, as also a considerable portion of the corpus callosum. The corpora striata were very slightly injected and softened, particularly that of the right side ; the lining membrane of the ventricles was not altered in colour. The brain, taken as a whole, excepting the parts mentioned, was natural.

*Spinal Marrow.*—On sawing the vertebræ, a considerable quantity of fluid blood gushed out the moment the interior of the canal was reached. It appeared to be perfectly flooded and engorged. The membranes were evidently thickened and highly vascular. The spinal marrow was not altered in appearance, but, if anything, softer than natural. The substance itself was not injected.

*Thorax.*—On inflating the lungs, the cells were permeate throughout. There was no appearance of hyperemia or inflammation of the substance, or of the lining membrane.

*The Heart* contained within the ventricles a thick coagulum of blood. The pericardium being cut through, about two ounces of serum escaped. The valves were healthy and the artery was free from deposits.

*Abdomen.*—The liver was perfectly engorged with blood ; an incision being made through its structure, the blood could be squeezed from it as from a sponge. The gall bladder was distended and contained a quantity of thick, black, bilious matter. The weight of the liver was 5½ pounds.

*The Kidnies* were congested, but otherwise healthy.

*The Spleen* was large, but contained little blood in comparison with the liver.

*The Intestines* contained a quantity of thin greenish matter. There were a few spots of ecchymoses in the lower two-fifths of the ilium. The small intestines contained a few large worms. The alimentary canal was otherwise throughout healthy. *The bladder* bore no mark of disease.

Such are the facts and history of one of the most malignant diseases that it has been my lot to witness.

I forbear making any remarks, as I have, I fear, trespassed upon your columns already too much.

Very respectfully,

Whiteville, Tenn., April, 1847. B. F. White.

(In reply to a note requesting further particulars, the following were received from Dr. White.—Eds.)

The disease made its appearance on the 25th day of February, near Hatchie river. The region of country extending along Hatchie, from Bolivar to Estanaula, was the only section that was affected. No case that I am aware of, occurred more than six miles out from the river. Some fifty cases that I know of, have occurred. The ratio of mortality in my practice, has been three-fourths out of the whole number. Every case that was taken with the extreme violent symptoms mentioned, died. Towards the last, the disease lost its malignancy to a certain extent, like all epidemics. However, the last two cases that I saw, died. The first of these demonstrated the futility of the antiphlogistic, with the sedative plan. In the second case, no time was afforded for any plan of treatment. The system received such a shock that it could never recuperate. The following were the notes taken at the time.

Miss Nancy E., aged 15 years, taken on Sunday evening, April 4th; complains of pain between the shoulders; spasmodic retraction of the posterior cervical muscles; subsultus tendinum; pain in the head and limbs; delirium, with intervals of consciousness; some fever, face flushed, bowels acted once during the day. April 5th, Monday morning, complains of pain of the stomach, with occasional sickness; slight delirium. Family gave an emetic; acted pretty thoroughly; delirium relieved; bowels evacuated; took a dose of calomel. I saw her first on Tuesday morning, 6th April, and found her in the following condition: head elevated on pillows; complains of an intense pain in the forehead; pulse hard and pupils contracted. On percussing the spinous processes, there was found to be tenderness, more particularly over the 2d and 3d dorsal vertebræ; tenderness on pressure over the right hypochondriac region; tongue covered with a thick yellow fur, fissured and dry; breath offensive. Bled her to ℥xvi, took ten ounces of blood by cups from the spine. A blister to extend from the neck, over the spine to the sacrum. Proto-chlor. hyd. grs. x, acetate morph. gr. i, for a powder. A cold douche over the head. She has now fallen to sleep. To take sulph. magnesia, 1 oz. in four hours, should she be not evacuated. Wednesday, 7th. I saw her this morning at 2 o'clock; still complains of pain in the head; pulse 75; bowels have acted freely; rational; no subsultus; pupils still contracted, Ordered a cold dash over the head, venesection to ℥xviii; to take proto-chlor. hydr. grs. x, acet. morph. gr. i, followed in four hours by a dose of sulph. magnesia. In my absence, a professional friend was called at 2 o'clock, P. M. He prescribed a dose of calomel with morphia. I saw her at ten o'clock at night. Head relieved, very little fever, small red spots, not unlike ecchymoses have appeared under the left eyelid, and over the body. To have two drops croton oil, repeated in two hours, should the bowels not act freely. Thursday, 9 o'clock, 8th April, complains of her head, great restlessness, sighing; pulse 100. Bowels were evacuated freely, discharges dark and very offensive. Stertorous breathing came on, and death took place at 12 o'clock.

In reviewing this case, you probably may be led to believe that the opium brought on the stertorous breathing, and that the treatment was sufficient to have produced death. I have seen precisely the same thing occur where no narcotic was given. The fact is, that I had almost lost confidence in any therapeutical agent. In those extreme cases, nothing appeared to be of any service. If any remedy is of any service, it is most unquestionably the preparations of opium.

I may remark, that in some of the cases that survived, the hearing was not restored for some time. The function of taste also, was lost. In one little child I recollect that he could not tell any thing bitter from any thing sweet. Every thing was devoured with the same gusto.

Yours, &c.,

Whiteville, May 18th, 1847. B. F. White.

To the Editors of the New Orleans Medical and Surgical Journal.

Gentlemen:—Conceiving it to be the duty of every medical philanthropist to contribute his mite towards the advancement of a science whose object is the relief of human suffering and prolonging life, I transmit to you for publication, if deemed worthy, the following observations upon a disease of most extraordinary character, which has lately prevailed in our city, to a moderate extent, but with great fatality. I submit them as taken from the bed-side of the sick, leaving the readers of your Journal to draw their own conclusions. When the disease first made its appearance, with what industry and zeal did the physicians of our city search the medical periodicals for half a century back, in order that they might receive some light as an inheritance from the experience of our predecessors, to aid them in the diagnosis and treatment of this fatal malady, but in vain; hence, I offer no other apology for this communication, hoping it may serve in a slight degree as a beacon, to aid some traveller through the dark mazes of our profession, and to shun the errors which have often prevented success.

The disease of which I speak, I shall call *myelitis petechialis*, as the meaning of the terms will convey an idea of the most prominent symptoms.

The patients were attacked with groaning, muttering delirium, chilly sensations, pallid countenance, extremities cold; which symptoms were soon followed by roving restlessness, flushed countenance, frequent pulse, expression of the eye wild and frantic, surface of the body hot and dry, violent screaming when touched or spoken to, unable to answer questions correctly, to locate the pain or point out the suffering organs; whilst sleeping, alternate pallor ond flushings of the countenance were often observed, and violent inflammation of the right or left eye was not an unfrequent occurrence. This condition was followed on the second or third day of the disease with symptoms of tetanus, the spinal muscles being very much contracted and rigid; in some instances to such an extent that the patients were enabled to swallow even fluids with much difficulty, accompanied with a loss of power in either the right or left upper and lower extremities, followed by convulsions of great severity, which would be excited or called into action by touching or raising the inferior extremities as quick as the operations of a galvanic apparatus. During the existence of this disturbed state of the nervous organism there was no evidence of lesion existing in the biliary or digestive organs, but great activity of the urinary secretions, being unnaturally copious and pellucid.

There was no uniform rule for the appearance of the various symptoms in this disease; in some instances the tetanic symptoms were observed as early as the first day of the attack, and in other cases as late as the tenth. In many of the violent cases, within six hours after the patients were taken ill, *petechiæ* of large size made their appearance upon the arms, over the eyelids and upon the inferior extremities, as if a violent hemorrhagic effort had been made and the blood arrested under the cuticle. These *petechiæ* disappeared on the 4th or 5th day, if the patients were not sooner cut off. In the most violent cases, death ensued on the 5th day of the disease; in other instances they survived from thirty to fifty days; but few cases recovered after the symptoms of tetanus presented themselves.

In consequence of a doubt existing in regard to the pathology of the disease, the treatment was pursued with a cautious and timid hand. The stimulating and anodyne course of treatment did not avail, and bloodletting was equally unsuccessful. The course which was of most promise, was of a mixed character. In vigorous constitutions, blood-letting in the onset of the attack, followed by an active mercurial cathartic, was of great service. If the patient should be of a more delicate constitution, cupping the whole length of the spine and early vesication by the use of the newly invented blistering tissue, or the unguent cantharides, would be preferable to the general bleeding; revulsives to the extremities and iced cloths to the head, together with the internal exhibition of the following mixture, greatly aided in advancing the case.

℞ Pulv. gum. camph. ʒj.

Potassio tart. antimonii, grs. ij.

Mucilage gum. acaciæ, ℥vi. M.

Give one tablespoonful every two hours. An enema composed of ʒij. spir. terebinthinæ, ʒij. tr. assafœtida and a half pint of thin starch was administered morning and evening, with a view of tranquillizing nervous distress, and keeping the bowels gently open.

After the violence of the disease had abated, the most beneficial tonic for relieving the inertia of the nervous system, which occurred in every instance of recovery, I found to be the following.

℞ Iod. ferri. ℈j.

Iodine, grs. viij.

Iod. potassæ, ʒij.

Syr. sarsapar. ℥iv.

Give one teaspoonful every four hours in a little water. This prescription should be continued for some days, unless found to produce gastric distress; then some mild bitter vegetable infusion should be substituted.

The pathology of this disease may be stated in a few words. The brain and spinal marrow being the great centre of the nervous system, any injury done to its substance by mechanical violence or otherwise, must necessarily derange the functions of all of the organs of the human machinery which depend upon their sane condition for the healthful elimination of the vital fluids and the depurative processes that are necessary to keep the organism in a condition suitable for the purposes of maintaining the proper operations of the whole; and when such important functions are so interfered with, the tendency is consequently to a cessation of that nervous power or excitation which is necessary to carry on the operations of vitality.

In the disease in question, there is a positive injury existing within the spinal canal; congestion and inflammation of the spinal marrow and its membranes, which, if not speedily arrested, terminates in softening of its substance, which must be necessarily fatal. This condition of the spinal marrow has been produced by the sudden operations of meteorological changes made upon the nervous expansions of the skin, which impressions are transmitted to the opposite extremity of the nervous apparatus through the medium of the nerves themselves, they being the natural conductors of sensations, at which point morbid action is excited and continued until lesion takes place, if not prevented by timely treatment. The pathology of nervous diseases has been too much neglected; the operations of the nervous system are much modified by the electrical condition of the atmosphere, and the treatment of this particular class of diseases will be unsatisfactory until more attention shall be paid to this particular study. The nervous system is more involved in the diseases of our climate than has been conceived of by medical writers. In intermittent fever we may bleed, blister and give alteratives, and we cannot cure the disease until we administer some remedy that will break up the morbid chain of nervous action, and keep up a proper equilibrium of the vascular system. What is the first organ upon which diseased action locates itself in the fevers of our miasmatic district; is it not the expansions of the nervous system? Do we not see shivering, and manifest derangement of nervous action before congestions of the spleen, liver and other organs take place, and the consequences attendant upon such conditions? Should the energy of the nervous system be of sufficient force, the equilibrium of the circulation would be maintained and no congestions could ensue.

B. J. Hicks, M. D.

March 31st, 1847. Vicksburg, Miss.

(In reply to a note requesting farther particulars, the following letter was received from Dr. Hicks.—Eds.)

Vicksburg, May 11th, 1847.

Gentlemen:—Yours of the 7th instant, has just come to hand.—The disease I gave you a partial account of under the head of *myelitis petechialis*, made its appearance in our city and its vicinity about the 10th of January last, and continued until the close of March; during which time between forty and fifty cases occurred, at least one-half of which proved fatal. No case to my knowledge recovered after decided symptoms of tetanus presented themselves.

Only one opportunity occurred in my practice of making a post-mortem examination, which, on account of press of business, was a hurried one, and consequently no organ was examined, except the brain and spinal marrow.

The cerebrum and cerebellum showed no symptoms of inflammation. The medulla oblongata and upper portion of the spinal marrow presented dots of blood when cut into, and the meninges of the same were found to be highly injected. I regret that I did not make a more perfect examination of this case, as no subsequent opportunity presented of investigating the pathology of this interesting disease. Yours, &c.

B. J. Hicks.

